# A rapid antigen test to detect adenosine deaminase 2 (ADA2) in biological fluids and its application in clinical diagnostics

**DOI:** 10.3389/fimmu.2025.1633219

**Published:** 2025-08-27

**Authors:** Weiping Yang, Qing Zhou, Jose M. Porcel, Jun Wang, Chengxiang Wu, Andrey V. Zavialov

**Affiliations:** ^1^ International Center for Aging and Cancer (ICAC), Hainan Medical University, Haikou, China; ^2^ Henan IVD Biotechnology, Zhengzhou, China; ^3^ Life Sciences Institute, Zhejiang University, Hangzhou, China; ^4^ Pleural Medicine Unit, Department of Internal Medicine, Arnau de Vilanova University Hospital, Lleida, Spain; ^5^ Tulane National Primate Research Center, Tulane University, Covington, LA, United States; ^6^ Turku Center for Biotechnology, University of Turku, Turku, Finland; ^7^ Guangzhou Institute of Pediatrics, Guangzhou Women and Children’s Medical Center, Guangzhou Medical University, Guangzhou, China

**Keywords:** adenosine deaminase 2, ADA2 deficiency, dada2, pleural tuberculosis, rapid antigen test

## Abstract

**Introduction:**

Saliva biomarkers provide a convenient and noninvasive method for diagnosing immune and genetic disorders. One such biomarker is adenosine deaminase 2 (ADA2), an enzyme that converts adenosine into inosine and is elevated in cancer and immune diseases. Currently, ADA2 activity can be used to diagnose pleural tuberculosis. Recently, we developed an ELISA assay that detects ADA2 in saliva. This test has been successfully employed to diagnose adenosine deaminase 2 deficiency (DADA2) and potentially to identify individuals with head and neck cancer. DADA2 is a rare genetic disease that can lead to systemic vasculitis, early onset stroke, bone marrow failure, and/or immunodeficiency. A rapid, simple, and affordable point-of-care (POC) test would significantly aid in diagnosing DADA2 and facilitate appropriate treatment.

**Methods:**

In this study, we created a novel colloidal gold-based immunochromatographic strip (ICS) containing polyclonal antigen-purified anti-ADA2 antibodies. The ADA2 rapid antigen test (RAT) was calibrated precisely to detect ADA2 in the saliva of healthy individuals, while those with ADA2 deficiency received negative results.

**Results:**

The test successfully confirmed DADA2 in nine patients with different pathogenic mutations in ADA2, while 58 of 59 healthy donors tested positive for ADA2.

**Discussion:**

The ADA2 rapid antigen test reliably screens patients with autoimmune disorders, accurately diagnosing DADA2. This test can also be used to diagnose pleural tuberculosis or other diseases with significantly elevated ADA2 levels.

## Introduction

Adenosine is a crucial molecule that regulates the cellular responses to activation signals. It binds to and activates adenosine receptors expressed in most cells, modulating their activity (1). Adenosine also affects gene expression by inhibiting enzymes necessary for protein and DNA methylation (2). Adenosine deaminases convert adenosine and deoxyadenosine to inosine and deoxyinosine, thereby regulating their concentrations (3). ADA1 and ADA2 are two adenosine deaminases in humans, and changes in ADA activity are associated with pathological conditions, such as immune disorders and cancer (4–6). ADA activity is used as a marker for the diagnosis and prediction of such disorders. For example, increased ADA2 activity has been used to diagnose pleural tuberculosis (7, 8). We developed ELISA-based assays for quantitative ADA2 detection in biological fluids that can diagnose head and neck cancers and ADA2 deficiency (DADA2) using saliva samples (5). DADA2 is a rare genetic disorder that affects more than 35,000 individuals worldwide (9). DADA2 patients have different mutations in the ADA2 gene, resulting in a broad clinical spectrum (10). While vasculitis and polyarteritis nodosa are associated with DADA2, patients also display hematologic abnormalities, such as pure cell aplasia and bone marrow failure (11). Mild hypogammaglobulinemia and immunodeficiencies with recurrent infections have also been observed. Early diagnosis and treatment are necessary to minimize organ damage and morbidity. However, rapid assays for ADA2 detection are not commonly available in clinics. The existing commercial kits primarily detect ADA activity in biological samples, including saliva. (12). Additionally, having both ADA1 and ADA2 in biological fluids, which have different catalytic properties, makes it difficult to compare the results of different ADA assays. (5). A standard HRP ELISA or ELISA based on the ADA2 activity is a suitable alternative, enabling quantitative measurement of ADA2 concentration (13). However, both the kits for ADA activity detection and ELISA tests are costly and require extra equipment to perform the tests. We describe a rapid, easy-to-use antigen test (RAT) that detects the enzyme in saliva, enabling the diagnosis of DADA2 deficiency at the point of care. This test can be used to screen patients, both in hospitals and at home. Additionally, we demonstrate that this test could potentially be used to diagnose other diseases, such as pleural tuberculosis, where ADA2 levels are significantly elevated.

## Materials and Methods

### DADA2 patients

The subjects were evaluated under a protocol (Protocol 2021-IRB-172) approved by the respective institutional review boards and provided written informed consent, including consent for publication. Pathogenic ADA2 mutations were identified in 9 DADA2 cases through Sanger sequencing. Among 9 genetically confirmed DADA2 patients, the median age was 18 years (interquartile range, 13–29 years), with 3 females included.

### TB patients

After receiving approval from the local ethics board (CEIC 1868), we randomly selected 41 patients with pleural tuberculosis (TB) and 48 patients with non-TB effusions from a biobank and database (IRBLleida Biobank B.0000682) maintained prospectively by the University Hospital Arnau de Vilanova in Lleida, Spain.

### Diagnostic criteria

A diagnosis of TB effusion is confirmed if auramine staining or cultures of pleural fluid, sputum, or pleural biopsy specimens are positive or if granulomas are present in the parietal pleura. TB was confirmed through high pleural fluid ADA levels (>35 U/L), negative cytology results, and effusion resolution following anti-TB treatment. The effusion is categorized as malignant if malignant cells are detected in the cytological examination of the pleural fluid or biopsy samples. Parapneumonic effusion refers to any effusion associated with bacterial pneumonia, which either resolves only with antibiotics (uncomplicated) or requires chest tube drainage (complicated). Other causes of pleural effusion are determined using well-established clinical criteria.

### Measuring ADA activity in pleural effusion

Pleural fluid samples obtained during thoracentesis were collected in 5 mL sterile heparinized tubes for immediate routine biochemical analysis, including ADA. Total ADA activity was determined using an automated spectrophotometric method (Roche Diagnostics, Barcelona, Spain).

### ADA2 measurement in pleural fluid by ELISA

ADA2 concentration in frozen pleural fluid samples collected during thoracentesis was measured using ADA2 ELISA (5, 13). ELISA plates (Greiner Bio-One) were coated overnight at 4°C with 100 μL of 5 μg/mL rabbit anti-ADA2 polyclonal antibodies in PBS containing 0.02% NaN_3_. After washing the plates three times with 200 μL PBS-Tween 20 buffer and blocking with 2% BSA in PBS containing 0.02% NaN_3_ for 1 hour, 100 μL of recombinant ADA2 standards diluted in PBS with 10% FBS or pleural fluid samples diluted in PBS with 0.02% NaN_3_ were added to the wells. The plates were incubated for 1 hour at room temperature on a shaker. Subsequently, the plates were washed three times with 200 μL PBS-Tween 20, and 100 μL of 2 mM adenosine in 20 mM Tris-HCl (pH 6.8), 10 μM ZnCl_2_, and 0.02% NaN_3_ was added. The plates were incubated at 37°C for 16–24 hours. The reaction was stopped by transferring 20 μL of the reaction mixture into a UV-transparent plate (Corning) containing 180 μL of water per well. The ratio of absorbance at 265 nm and 245 nm was measured using a Thermo Fisher Multiscan Go Reader. The ADA2 concentration in the samples was determined from a standard curve generated with the recombinant protein.

### Sequencing

Sanger sequencing of the 10 coding exons of ADA2 was performed as previously described (9).

### Saliva samples

Healthy donors (n=59) and nine patients with confirmed DADA2 participated in this study. Healthy volunteers and patients self-collected saliva samples, and a rapid antigen test was used to detect ADA2 in saliva. The median age of healthy donors was 33 years, with an interquartile range of 21 to 50 years.

### ADA2 cloning, expression, and purification

ADA2 was expressed and purified according to the method described by Kaljas et al. (14) with minor modifications. To generate a cell line stably overexpressing human ADA2 through transduction with a lentiviral vector, we amplified the open reading frames (ORFs) of the genes by PCR using specific primers: (F) 5’-ATCTCGAGCCACCATGTTGGTGGATGGCCCATCTG-3’ and (R) 5’-TAGGATCCTCACTTTGTAGCCACATCTGC-3’ (hADA2). The PCR products were then subcloned into the pCR2.1-TOPO plasmid, excised by the restriction digest using XhoI and BamHI, and finally, ligated into an XhoI/BamHI-digested self-inactivating (SIN) transfer plasmid (pHR-cPPT-hB7-SIN) (15).

To generate lentiviral vectors, HEK-293T cells were transfected with the lentiviral transfer plasmid containing the ADA2 gene, pCMV-VSV-G envelope, and pCMVΔR8.2 packaging plasmid using an optimized calcium phosphate method (16). The lentiviral vectors were then concentrated via ultracentrifugation from the conditioned medium of transfected HEK-293T cells. Concentrated lentiviral vectors were used to infect 293T cells. Finally, recombinant ADA2 was purified from the conditioned medium of lentivirus-transduced 293T cells using a specific protocol. The cell culture medium was diluted three times with water, and 1 M Tris-HCl (pH 6.8) was added to achieve a final concentration of 20 mM Tris-HCl (pH 6.8) and 0.02% NaN_3_. The Heparin HiTrap column (Cytivia) was equilibrated with buffer A (20 mM Tris HCl pH 6.8, 50 mM NaCl, 0.02% NaN_3_), and the diluted cell culture medium was applied to the column. The column was washed with buffer A until a stable UV baseline was achieved. The HiTrap Protein G column equilibrated in buffer A was attached to the heparin column to remove traces of IgG. ADA2 was eluted with 20 mM Tris-HCl, pH 6.8, 0.5 M NaCl, and 0.02% NaN_3_, and the fractions containing ADA2 activity were collected. The enzyme was concentrated using a 30 kDa Amicon Ultra Centrifugal filter. The concentrate was diluted ten times with 50 mM Tris-HCl (pH 8.0), 10 μM ZnCl2, and 0.02% NaN_3_ to achieve a final concentration of 5 mM NaCl. In the next step, the DEAE HiTrap Sepharose column was washed with 20 mM Tris HCl (pH 8.0), 10 μM ZnCl_2_, and 0.02% NaN_3_, and partially purified ADA2 was applied to the column. The flow-through was collected and concentrated using a 30 kDa Amicon Ultra Centrifugal filter. The concentrate was applied to a Superdex 200 column (Cytivia) equilibrated with 1xPBS, 10 μM ZnCl_2_, and 0.02% NaN_3_. After gel filtration, the fractions containing ADA2 activity were pooled, and the recombinant protein was used to immunize rabbits.

### Polyclonal antibody production and purification

Rabbits were administered two doses of 1 mg recombinant ADA2 to induce the production of polyclonal antibodies. These antibodies were purified from rabbit serum using a 5 mL HiTrap Protein G affinity column manufactured by Cytiva. The column was prepared by equilibrating it with 1x PBS buffer, and the serum was then loaded onto it. The column was washed with 1xPBS buffer until a stable UV baseline was achieved. The antibodies were then eluted from the column using 0.1 M Glycine buffer at pH 2.7 and immediately neutralized with a 1:10 volume of 1 M Tris-HCl buffer. The buffer was then exchanged with 1x PBS, 0.02% NaN_3_, using a 30 kDa Amicon Ultra Centrifugal filter. To isolate ADA2-specific antibodies, recombinant ADA2 was chemically biotinylated using EZ-Link NHS-PEG4-Biotin (Thermo Fisher) and purified on a HiTrap desalting column equilibrated with 1x PBS. Biotinylated ADA2 was then bound to a HiTrap Streptavidin column in 1xPBS. The anti-ADA2 antibodies were then purified on a column loaded with biotinylated ADA2. The binding buffer was 1xPBS, and the elution buffer was 0.1 M Glycine buffer containing 200 mM NaCl at pH 2.7. To bring the pH to neutral, a 1 M Tris-HCl buffer was used in a 1:10 v/v ratio, and the buffer was exchanged with 1x PBS, 0.02% NaN3, using a 30 kDa Amicon Ultra Centrifugal filter.

### Preparation of colloidal gold anti-ADA2 antibody conjugates

The colloidal gold solution was prepared by adding 100 mL of 1% HAuCl_2_ to a clean glass beaker (250 mL) and stirring using a magnetic stirrer heater. The mixture was then heated to 100°C. Next, 0.7 mL of 1% sodium citrate (Na_3_C_6_H_5_O_7_·2H_2_O) was slowly added to the HAuCl_2_ solution, followed by continuous stirring for 20 minutes. Subsequently, the solution was cooled to room temperature. To this, 150 μg of antigen-purified antibodies against ADA2 was added to 10 ml of colloidal gold solution at pH 7.8, and the mixture was stirred for 30 min at room temperature. Aqueous bovine serum albumin (BSA) (5% wt/vol; 2.5 mL) was added to block any endogenous colloidal gold reactivity. The resulting mixture was centrifuged at 17,000 g and 4°C for 30 min to remove unbound antibodies. Finally, the pellet was resuspended in 2 mL of resuspend solution (0.01 M Tris-HCl, pH 8.0, containing 0.1% PEG6000 and 0.2% BSA), and the extent of conjugation between colloidal gold and antibodies was assessed using UV-visible (UV-Vis) spectroscopy.

### The immunochromatographic strip preparation

**Figure 1:**
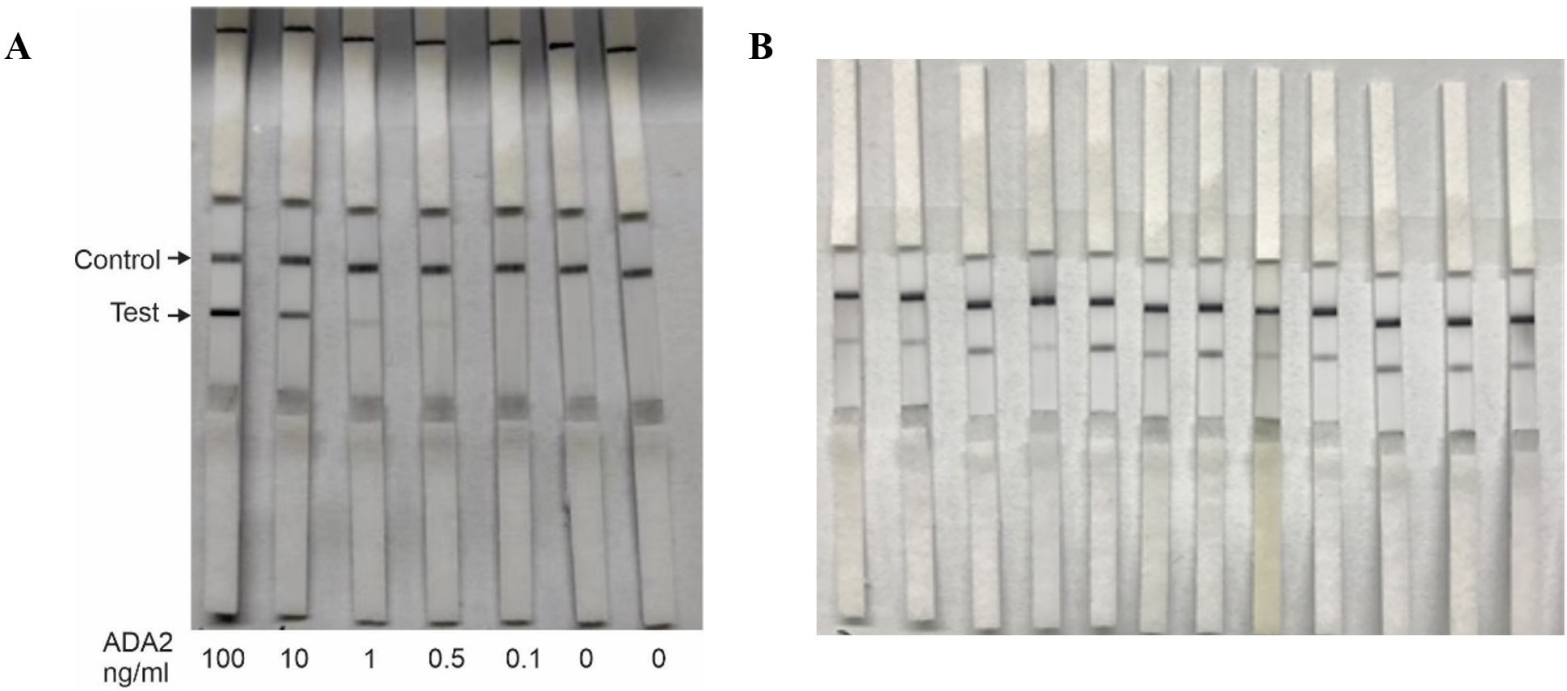
Lateral flow test strips for rapid detection of ADA2 in saliva. **(A)** Analytical sensitivity of the test. 55 μL of PBS buffer containing different concentrations of ADA2 (0–100 ng/mL) was applied to the lateral flow test strips, and the results were obtained after 15 min. **(B)** Saliva from healthy donors. Fifty-five microliters of saliva from healthy volunteers were applied to the lateral flow test strips, and the results were obtained after 15 minutes. The control line contains goat anti-rabbit antibodies.

Immunochromatographic strips (ICS) shown in **Figure 1** were prepared according to the following protocol. Initially, the sample pad was saturated with PBS solution (pH 8.5) containing 0.5% Tween 20 and 0.1% (w/v) BSA. Subsequently, the glass fiber paper was impregnated with a solution of gold-conjugated anti-ADA2 polyclonal antibodies (ADA2 pAbs). The sample pad was dried at 37°C for an hour and stored in a desiccator for future use. Next, diluted ADA2 pAbs and goat anti-rabbit IgG (1 mg/mL) were printed onto a nitrocellulose (NC) membrane using PBS containing 2% sucrose as the medium. After drying for 2 h at 37°C, the NC membranes were stored in a desiccator. The final assembly combined the absorption pad, nitrocellulose membrane, gold conjugate pad, and sample pad onto a plastic backplate in a dry room. These components were then cut into test strips, assembled into plastic cassettes, and sealed in aluminum foil bags.

### Saliva sample preparation and testing procedures

**Figure 2:**
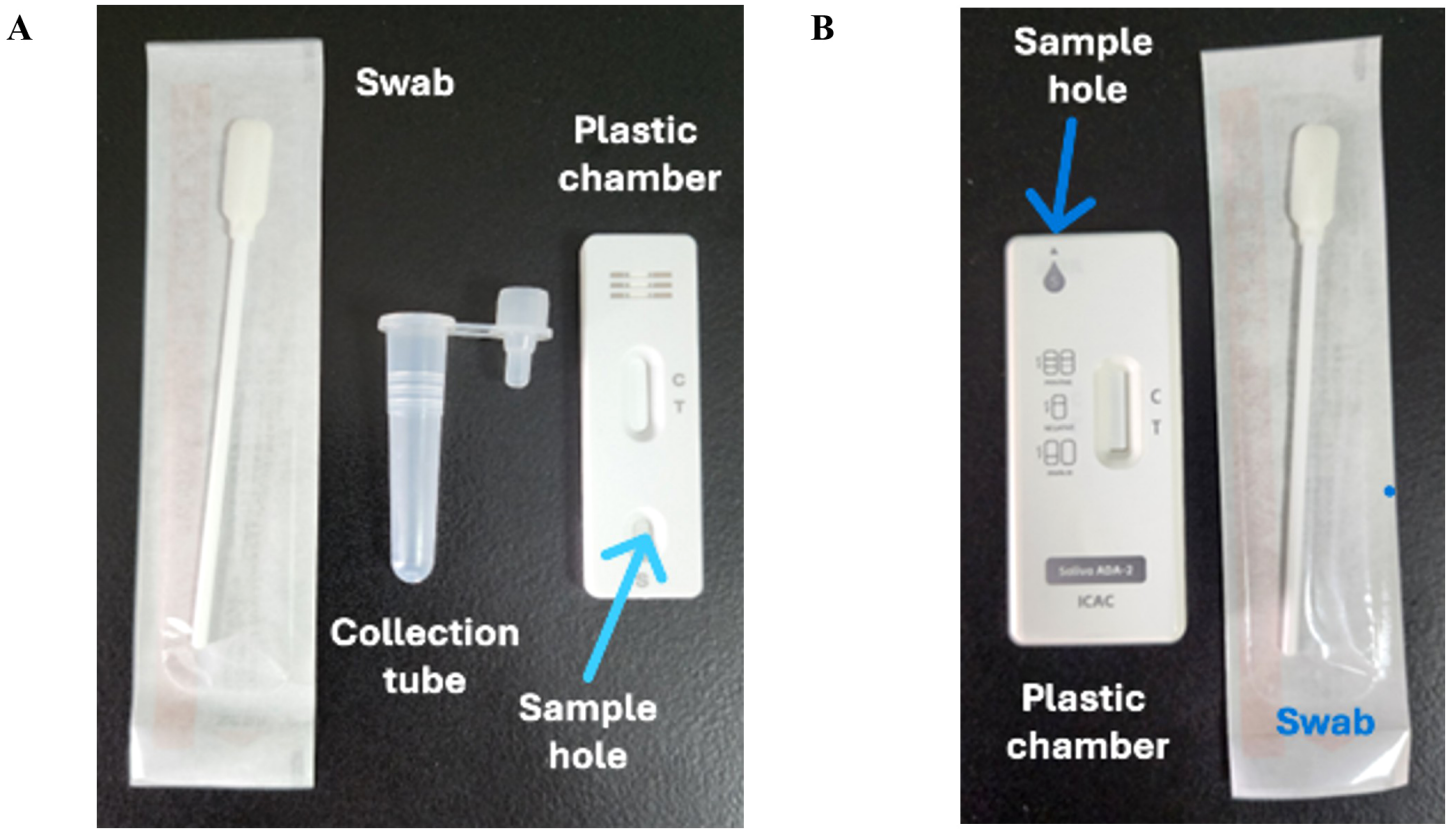
Design of rapid antigen test. **(A)** The test consisted of a swab, saliva collection tube, and plastic chamber with a lateral flow test strip. **(B)** The test consisted of a swab and a plastic chamber with a lateral flow test strip and a hole on top to insert the swab with the saliva sample. The control line contains goat anti-rabbit antibodies.

Saliva samples in the present study were collected at least one hour after food or fluid intake, during either morning or afternoon hours (9:00 AM to 5:00 PM). To collect saliva for testing, a sterile swab was rolled into the mouth for 90 seconds to increase absorption. There were two methods of loading the saliva samples onto the strip. The first method involves inserting the swab with saliva directly into a plastic chamber, squeezing the saliva, and then inserting the detection strip (**Figure 2B**). Alternatively, saliva can be squeezed into a plastic tube, and 3 drops (100 μL) of the collected saliva can be applied to the sample pad (**Figure 2A**). The results were read within 15 minutes in both cases to determine the outcome.

### Testing the pleural fluid samples by RAT

The samples were diluted 750 times with PBS to match the sensitivity of the RAT assay, and 100 μl of the diluted samples were applied to the RAT DADA2 test. The results were read after 15 minutes of incubation.

## Results

**Figure 3:**
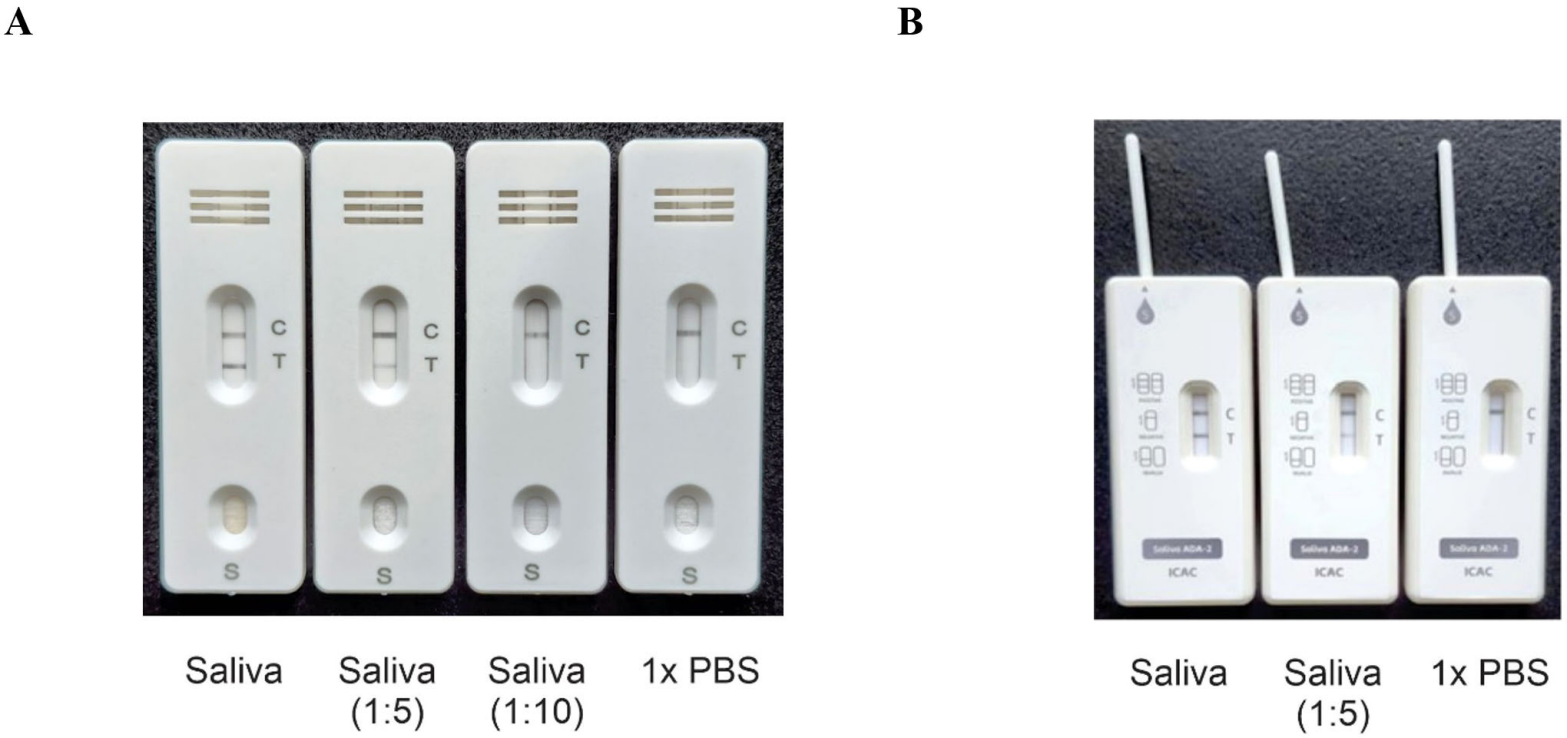
Typical test results. **(A)** Three drops of the sample (100 μl) containing undiluted saliva and saliva diluted with PBS were applied to the sample pad, and the result was obtained after 15 minutes. **(B)** A swab with undiluted saliva, saliva diluted with PBS, or PBS was inserted into the plastic chamber, and the result was obtained after 15 minutes. The control line contains goat anti-rabbit antibodies.

Previously, we used ELISA-based assays to demonstrate that the concentration of ADA2 in the saliva of DADA2 patients is significantly lower than that in healthy subjects (5). To ensure the sensitivity of the test, we designed lateral flow test strips with a limit of detection of 0.5 ng/mL ADA2, which is the lowest concentration of ADA2 in the saliva of healthy donors (5). As shown in **Figure 1A**, a faint band was still visible when a solution containing 0.5 ng/mL ADA2 was applied to the sample pad on the strip. Conversely, no ADA2 was detected when the concentration was 0.1 ng/mL. Since the concentration of ADA2 in the saliva of DADA2 patients is at least 10 times lower than that of healthy controls (5), the test is expected to yield negative results with saliva samples from DADA2 patients, unlike those from healthy donors (**Figure 1B**). The testing of saliva samples from healthy donors revealed the detection of ADA2 in saliva, except for one donor (**Table 1**), with an assay specificity of 98.3%. Two rapid antigen kits were designed for further testing. The first kit contained a sterile swab for collecting saliva from the mouth, a tube for squeezing saliva into, and a chamber for applying 3 drops onto the sample pad (**Figure 2A**). This kit also performs well when saliva is collected directly into a tube without using a swab. The second test system contained a swab for collecting saliva from the mouth, which was inserted into a plastic chamber, and the sample was directly applied to the lateral flow test strip (**Figure 2B**). Both designs yielded similar results when tested with saliva samples from healthy donors or with 1× PBS buffer as a control (**Figure 3**).

**Table 1:** The summary of the test results for the healthy volunteers.

	Number	Age	Positive for ADA2	Negative for ADA2	Specificity
Healthy donors	59	33 (21-50)	58	1	98.3%

Data are expressed as medians (interquartile range).

To test the efficacy of RAT ADA2 in diagnosing DADA2, we collected saliva samples from nine patients with DADA2 who had various mutations or deletions in the ADA2 gene (**Table 2**). The test was conducted in both the laboratory and at home. Our results showed that none of the nine DADA2 patients had detectable ADA2 in their saliva, indicating that RAT ADA2 could be used as a screening tool for patients exhibiting symptoms of DADA2 (10).

**Table 2:** **S**ummary of test results for DADA2 patients.

Number	Mutation	Negative saliva testing for ADA2
1	Y453C and Y453C	Yes
2	Y453C and Y453C	Yes
3	G47R and G47R	Yes
4	R169Q and R131Sfs*52	Yes
5	exon7 deletion and exon7 deletion	Yes
6	T33Nfs*29 and G358R	Yes
7	Arg169Gly and Arg169Gly	Yes
8	Arg169Gly and Arg169Gly	Yes
9	G47W and R49Gfs*4	Yes

"*" denotes a premature stop codon, indicating that the altered protein sequence terminates early due to the frameshift.

**Figure 4:**
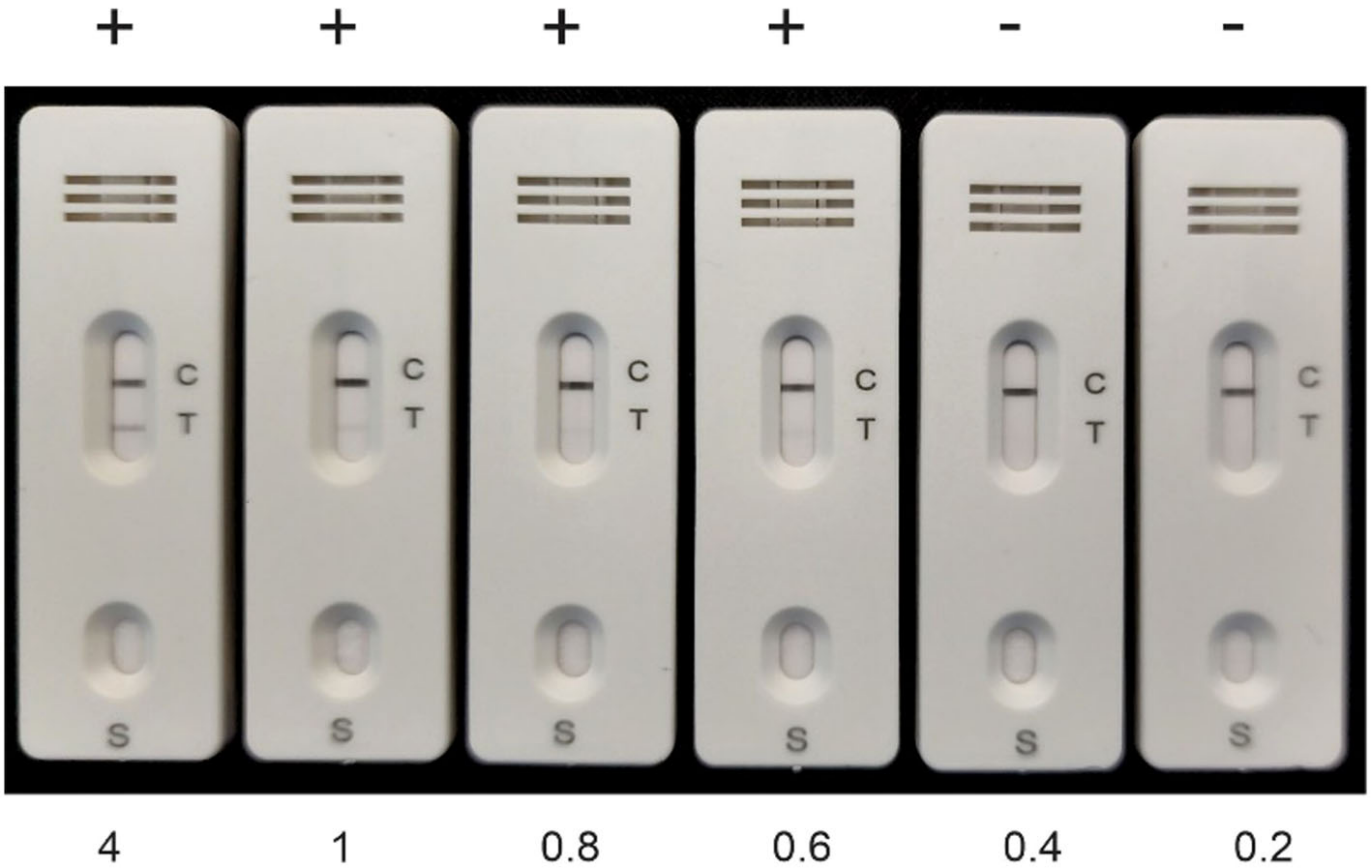
Calibration of the rapid antigen test with ADA2. Recombinant ADA2 in 100 μL of buffer containing different concentrations of ADA2 (ng/mL) was applied to the RAT, and the results were obtained after 15 min. The control line contains goat anti-rabbit antibodies.

**Figure 4** illustrates that the test can differentiate between 0.6 ng/mL and 0.4 ng/mL of ADA2, resulting in positive and negative outcomes, respectively. Considering the data on ADA2 concentration in the pleural fluid of patients with pleural tuberculosis, as presented in **Supplementary Table 1**, we anticipate that the test will have high specificity in diagnosing TB (7). According to our data, when pleural fluid is diluted 750 times, 100% of patients with tuberculosis (TB) will test positive. In contrast, 10.6% of non-TB patients (5 out of 47) will yield false positives or indeterminate results, where the band is very faint. However, clinical testing is necessary to validate RAT ADA2 for the diagnosis of tuberculosis (TB).

## Discussion

Our study introduces a novel rapid antigen test (RAT) for detecting adenosine deaminase 2 (ADA2) in saliva and pleural fluid, providing a convenient and noninvasive method for the preliminary diagnosis of DADA2 and potentially pleural tuberculosis (TB). The ability to screen for ADA2 deficiency at the point of care significantly enhances diagnostic efficiency, offering clinicians and patients a reliable and accessible tool for early detection. The ADA2 RAT demonstrated high specificity and sensitivity when tested on saliva samples from healthy individuals and patients with DADA2, albeit in only nine patients. With a limit of detection of 0.5 ng/mL ADA2, the test could accurately distinguish between individuals with normal ADA2 expression and those with ADA2 deficiency. These results align with prior ELISA-based assessments that confirmed significantly lower ADA2 concentrations in the saliva of DADA2 patients compared to healthy donors (5). Given the implications of early-onset stroke, systemic vasculitis, immunodeficiency, and bone marrow failure, a rapid screening method can play a crucial role in identifying patients who require immediate therapeutic intervention. Additionally, our findings suggest that the test could be adapted to detect pleural tuberculosis, as pleural fluid ADA2 levels are significantly elevated in TB patients compared to non-TB individuals. While our data support the feasibility of RAT ADA2 for TB screening, clinical validation studies are needed to confirm its accuracy in diverse patient populations. If validated, the test could provide a cost-effective alternative to traditional ADA2 assays used in TB diagnosis, facilitating early treatment decisions in resource-limited settings. One of the key advantages of this immunochromatographic strip test is its user-friendly design, making it suitable for both clinical and home-based applications. The ability to detect ADA2 in self-collected saliva samples ensures accessibility for patients, thereby reducing their dependence on laboratory-based diagnostics. Both test kits—one incorporating a saliva collection tube and the other using a direct swab application—produced comparable results, highlighting the flexibility in test administration. This adaptability is particularly valuable for pediatric patients and individuals in remote areas, where conventional laboratory testing may not be readily available. Moreover, home-based testing facilitates early disease monitoring, allowing patients and caregivers to track ADA2 expression levels over time. The potential expansion of RAT ADA2 to monitor immune disorders, head and neck cancer, and large granulocyte leukemia enhances its applicability in personalized healthcare, providing patients and clinicians with a tool for real-time decision-making. While the ADA2 RAT has demonstrated high specificity in DADA2 diagnosis, particular challenges remain. One healthy donor produced a false-negative result, suggesting that minor fluctuations in ADA2 expression might affect test sensitivity. Further refinement of the test strip’s detection threshold could enhance accuracy, ensuring minimal occurrences of false positives or false negatives. For TB screening, a larger clinical validation study is necessary to confirm the diagnostic performance of RAT ADA2 across diverse populations. Standardizing saliva collection methods and refining test strip calibration could optimize reproducibility in different healthcare settings. Additionally, expanding ADA2 RAT applications to identify immune disorders and cancers may require comparative studies alongside traditional ELISA-based assays to establish the broader clinical utility of these methods.

## Conclusions

The development of an ADA2 rapid antigen test represents a significant advancement in point-of-care diagnostics, particularly for the diagnosis of DADA2 and the screening of pleural tuberculosis. Its ability to detect ADA2 in saliva, combined with high sensitivity and ease of use, makes it a promising tool for early disease detection. With further validation in screening for DADA2 in patients with primary immunodeficiencies, the ADA2 RAT could pave the way for accessible, home-based healthcare solutions, improving disease outcomes through timely diagnosis and intervention.

## Data Availability

The original contributions presented in the study are included in the article/**Supplementary Material**. Further inquiries can be directed to the corresponding authors.

## References

[1] Eltzschig HK Sitkovsky MV Robson SC. Purinergic signaling during inflammation. N Engl J Med. (2012) 367:2322–33. doi: 10.1056/NEJMra1205750, PMID: 23234515 PMC3675791

[2] Zhulai G Oleinik E Shibaev M Ignatev K. Adenosine-metabolizing enzymes, adenosine kinase and adenosine deaminase, in cancer. Biomolecules. (2022) 12(3):418. doi: 10.3390/biom12030418, PMID: 35327609 PMC8946555

[3] Dong L Luo W Maksym S Robson SC Zavialov AV. Adenosine deaminase 2 regulates the activation of the toll-like receptor 9 in response to nucleic acids. Front Med. (2024) 18(5):814–30. doi: 10.1007/s11684-024-1067-5, PMID: 39078537

[4] Antonioli L Colucci R La Motta C Tuccori M Awwad O Da Settimo F. Adenosine deaminase in the modulation of immune system and its potential as a novel target for treatment of inflammatory disorders. Curr Drug Targets. (2012) 13:842–62. doi: 10.2174/138945012800564095, PMID: 22250650

[5] Luo W Dong L Chen F Lei W He L Zhou Q. ELISA based assays to measure adenosine deaminases concentration in serum and saliva for the diagnosis of ADA2 deficiency and cancer. Front Immunol. (2022) 13:928438. doi: 10.3389/fimmu.2022.928438, PMID: 35967411 PMC9366848

[6] Dong L Lu B Luo W Gu X Wu C Trotta L. Intracellular concentration of ADA2 is a marker for monocyte differentiation and activation. Front Med. (2025) 19(2):359–75. doi: 10.1007/s11684-024-1110-6, PMID: 39832022

[7] Porcel JM. Pleural fluid biomarkers: beyond the Light criteria. Clin Chest Med. (2013) 34:27–37. doi: 10.1016/j.ccm.2012.11.002, PMID: 23411054

[8] Zemlin AE Burgess LJ Carstens ME. The diagnostic utility of adenosine deaminase isoenzymes in tuberculous pleural effusions. Int J Tuberc Lung Dis. (2009) 13:214–20., PMID: 19146750

[9] Zhou Q Yang D Ombrello AK Zavialov AV Toro C Stone DL. Early-onset stroke and vasculopathy associated with mutations in ADA2. N Engl J Med. (2014) 370:911–20. doi: 10.1056/NEJMoa1307361, PMID: 24552284 PMC4193683

[10] Lee PY Davidson BA Abraham RS Alter B Arostegui JI Bell K. Evaluation and management of deficiency of adenosine deaminase 2: an international consensus statement. JAMA Netw Open. (2023) 6:e2315894. doi: 10.1001/jamanetworkopen.2023.15894, PMID: 37256629

[11] Lee PY Aksentijevich I Zhou Q. Mechanisms of vascular inflammation in deficiency of adenosine deaminase 2 (DADA2). Semin Immunopathol. (2022) 44(3):269–280. doi: 10.1007/s00281-022-00918-8, PMID: 35178658

[12] Franco-Martinez L Tecles F Torres-Cantero A Bernal E San Lazaro I Alcaraz MJ. Analytical validation of an automated assay for the measurement of adenosine deaminase (ADA) and its isoenzymes in saliva and a pilot evaluation of their changes in patients with SARS-CoV-2 infection. Clin Chem Lab Med. (2021) 59:1592–9. doi: 10.1515/cclm-2021-0324, PMID: 33908223

[13] Skaldin M Porcel JM Lamminmäki U Bielsa S Zavialov AV. Developing and validating anti-ADA2 single-chain antibodies coupled to alkaline phosphatase for diagnosing pleural tuberculosis [Original Research]. Front Immunol. (2025) 6:2025. doi: 10.3389/fimmu.2025.1646134 PMC12390807 40895545

[14] Kaljas Y Liu C Skaldin M Wu C Zhou Q Lu Y. Human adenosine deaminases ADA1 and ADA2 bind to different subsets of immune cells. Cell Mol Life Sci. (2017) 74:555–70. doi: 10.1007/s00018-016-2357-0, PMID: 27663683 PMC11107696

[15] Wu C Lu Y. High-titre retroviral vector system for efficient gene delivery into human and mouse cells of haematopoietic and lymphocytic lineages. J Gen Virol. (2010) 91:1909–18. doi: 10.1099/vir.0.020255-0, PMID: 20410313 PMC3052536

[16] Wu C Lu Y. Inclusion of high molecular weight dextran in calcium phosphate-mediated transfection significantly improves gene transfer efficiency. Cell Mol Biol (Noisy-le-grand). (2007) 53:67–74., PMID: 17531163 PMC2830788

