# Supplementary material for: A rapid antigen test to detect adenosine deaminase 2 (ADA2) in biological fluids and its application in clinical diagnostics
Source: Front Immunol. 2025 Aug 27;16:1633219. doi: 10.3389/fimmu.2025.1633219 (PMC12420330; doi:10.3389/fimmu.2025.1633219)
Supplement: Supplementary file 1 [file Table1.docx]

**Supplementary Materials**

**Supplementary Table 1.**  The use of the ADA2 RAT test for diagnosing pleural tuberculosis. The concentration of ADA2 in pleural fluid from patients with tuberculosis (TB), malignant pleural effusion (MPE), uncomplicated parapneumonic pleural effusion (UCPPE), complicated parapneumonic pleural effusion (CPPE), and heart failure (HF) was measured by ELISA (Luo et al., 2022; Skaldin et al., 2025). The results of ADA2 RAT are based on Figure 4 (1:750 dilution of pleural fluid). The ADA2 concentration should be interpreted as follows: concentrations above 450 ng/mL are considered positive, concentrations below 300 ng/mL are considered negative, and concentrations between 300-450 ng/mL are considered indeterminate.

| Number | Patient ID | Diagnosis | ADA2 ng/ml | Standard  deviation (SD) | RAT test  1:750 dilution |
| --- | --- | --- | --- | --- | --- |
| 1 | 14238214 | TB | 1071.18 | 18 | positive |
| 2 | 14213225 | TB | 768.33 | 7.92 | positive |
| 3 | 13961247 | TB | 973.92 | 1.17 | positive |
| 4 | 14043323 | TB | 932.25 | 63.57 | positive |
| 5 | 15234778 | TB | >1200 |  | positive |
| 6 | 13900301 | TB | >1200 |  | positive |
| 7 | 14243466 | TB | >1200 |  | positive |
| 8 | 14644626 | TB | 958.35 | 6 | positive |
| 9 | 14580494 | TB | 1001.52 | 10.95 | positive |
| 10 | 13949645 | TB | 966.36 | 27.24 | positive |
| 11 | 13917816 | TB | >1200 |  | positive |
| 12 | 13938526 | TB | 749.01 | 7.53 | positive |
| 13 | 14471287 | TB | 1080.24 | 1.98 | positive |
| 14 | 14433705 | TB | >1200 |  | positive |
| 15 | 639613 | TB | >1200 |  | positive |
| 16 | 636774 | TB | >1200 |  | positive |
| 17 | 559639 | TB | 368.7 | 4.95 | positive |
| 18 | 561821 | TB | 789.06 | 23.1 | positive |
| 19 | 626861 | TB | 656.73 | 28.23 | positive |
| 20 | 223087 | TB | 934.86 | 11.46 | positive |
| 21 | 350481 | TB | >1200 |  | positive |
| 22 | 250529 | TB | 806.79 | 23.94 | positive |
| 23 | 301058 | TB | 906.54 | 38.46 | positive |
| 24 | 250278 | TB | 1152.93 | 4.05 | positive |
| 25 | 307460 | TB | 1243.89 |  | positive |
| 26 | 462344 | TB | 1184.61 | 20.1 | positive |
| 27 | 248319 | TB | 558.36 | 17.55 | positive |
| 28 | 510414 | TB | 1023.48 | 49.83 | positive |
| 29 | 15182156 | TB | 725.7 | 2.7 | positive |
| 30 | 13934063 | TB | 922.71 | 15 | positive |
| 31 | 14870813 | TB | 440.64 | 0.36 | positive |
| 32 | 11853301 | TB | 559.5 | 23.7 | positive |
| 33 | 14320628 | TB | >1200 |  | positive |
| 34 | 14445046 | TB | 686.49 | 12.06 | positive |
| 35 | 13977620 | TB | 945.48 | 15.54 | positive |
| 36 | 614767 | TB | 826.29 | 46.26 | positive |
| 37 | 535482 | TB | 1167.36 | 19.65 | positive |
| 38 | 308030(P) | TB | 888.12 | 3.72 | positive |
| 39 | 616008 | TB | 633.09 | 5.13 | positive |
| 40 | 282086 | TB | >1200 |  | positive |
| 41 | 575665 | TB | >1200 |  | positive |
| 42 | 15327140 | UCPPE | 129.93 | 24.6 | negative |
| 43 | 14420403 | UCPPE | 157.41 | 11.43 | negative |
| 44 | 14083423 | UCPPE | 191.97 | 3.66 | negative |
| 45 | 13974275 | UCPPE | 132.45 | 0.51 | negative |
| 46 | 14298997 | UCPPE | 297.9 | 8.88 | negative |
| 47 | 14270824 | UCPPE | 89.82 | 1.38 | negative |
| 48 | 14144113 | UCPPE | 131.88 | 0.03 | negative |
| 49 | 14144806 | UCPPE | 281.1 | 11.31 | negative |
| 50 | 14051940 | UCPPE | 213.18 | 8.97 | negative |
| 51 | 13329994 | UCPPE | 80.52 | 7.32 | negative |
| 52 | 12783298 | UCPPE | 241.23 | 0.54 | negative |
| 53 | 14151382 | UCPPE | 107.01 | 0.09 | negative |
| 54 | 14183009 | UCPPE | 274.62 | 0.03 | negative |
| 55 | 13021186 | UCPPE | 319.83 | 17.82 | indeterminate |
| 56 | 11866270 | CPPE | 142.38 | 9.63 | negative |
| 57 | 14155337 | CPPE | 159.93 | 3.54 | negative |
| 58 | 15204473 | CPPE | 102.87 | 14.4 | negative |
| 59 | 14253111 | CPPE | 109.38 | 36.51 | negative |
| 60 | 14222090 | CPPE | 95.52 | 15.99 | negative |
| 61 | 13897781 | CPPE | 185.25 | 7.08 | negative |
| 62 | 14254277 | CPPE | 176.85 | 8.16 | negative |
| 63 | 14201448 | CPPE | 406.56 | 8.49 | indeterminate |
| 64 | 13933995 | MPE | 110.34 | 25.65 | negative |
| 65 | 14091151 | MPE | 193.56 | 5.52 | negative |
| 66 | 14092672 | MPE | 85.02 | 27.87 | negative |
| 67 | 13913816 | MPE | 85.95 | 8.64 | negative |
| 68 | 14245289 | MPE | 225.81 | 3.96 | negative |
| 69 | 14773058 | MPE | 179.67 | 3.69 | negative |
| 70 | 14106891 | MPE | 150.57 | 3.69 | negative |
| 71 | 14213174 | MPE | 78.15 | 1.8 | negative |
| 72 | 14285455 | MPE | 302.82 | 7.41 | negative |
| 73 | 14233221 | MPE | 272.49 | 11.34 | negative |
| 74 | 14236468 | MPE | 444.24 | 12.54 | indeterminate |
| 75 | 14029759 | MPE | 296.04 | 1.59 | negative |
| 76 | 14237664 | MPE | 433.8 | 15.69 | indeterminate |
| 77 | 12308797 | MPE | 224.94 | 3.84 | negative |
| 78 | 578760 | MPE | 535.08 | 2.58 | positive |
| 79 | 493522 | MPE | 274.14 | 15.21 | negative |
| 80 | 648985 | MPE | 351.42 | 2.52 | negative |
| 81 | 188721 | MPE | 190.35 | 2.4 | negative |
| 82 | 433090 | MPE | 54.39 | 5.88 | negative |
| 83 | 389093 | MPE | 23.43 | 21.96 | negative |
| 84 | 10376838 | HF | 98.13 | 29.1 | negative |
| 85 | 14100686 | Idiopathic | 138.57 | 15.84 | negative |
| 86 | 14232145 | Pericarditis | 40.83 | 2.91 | negative |
| 87 | 11977073 | Pericarditis | 96.06 | 15.36 | negative |
| 88 | 13895308 | Pericarditis | 175.53 | 3.75 | negative |

Luo, W., Dong, L., Chen, F., Lei, W., He, L., Zhou, Q., Lamy, T., & Zavialov, A. V. (2022). ELISA based assays to measure adenosine deaminases concentration in serum and saliva for the diagnosis of ADA2 deficiency and cancer. *Front Immunol*, *13*, 928438. <https://doi.org/10.3389/fimmu.2022.928438>

Skaldin, M., Porcel, J. M., Lamminmäki, U., Bielsa, S., & Zavialov, A. V. (2025). Developing and validating anti-ADA2 single-chain antibodies coupled to alkaline phosphatase for diagnosing pleural tuberculosis [Original Research]. *Front Immunol*, *Volume 16 - 2025*. <https://doi.org/10.3389/fimmu.2025.1646134>
